# Mitochondrial Genome Characteristics and Phylogenetic Analysis of *Fulmekiola serrata* (Kobus) (Thysanoptera: Thripidae)

**DOI:** 10.3390/ijms251910431

**Published:** 2024-09-27

**Authors:** Jiong Yin, Zhi-Ming Luo, Yin-Hu Li, Chang-Mi Wang, Jie Li, Rong-Yue Zhang, Hong-Li Shan, Xiao-Yan Wang, You-Qing Chen

**Affiliations:** 1 Institute of Highland Forest Science, Chinese Academy of Forestry, Kunming 650233, China; yinjiong@126.com; 2 Graduate School of Nanjing Forestry University, Nanjing 210037, China; 3 Sugarcane Research Institute, Yunnan Academy of Agricultural Sciences/Yunnan Engineering Research Center of Sugar Industry, Kaiyuan 661699, China

**Keywords:** sugarcane thrips, mitochondrial genome, phylogeny, gene rearrangement

## Abstract

Sugarcane thrips, *Fulmekiola serrata* (Kobus) (Thysanoptera: Thripidae), is a common foliar pest that infests sugarcane and is found throughout tropical and subtropical countries. In this study, we obtained and analyzed the complete mitochondrial genome of *F. serrata* for the first time and explored the phylogenetic relationships of the higher-order elements of Thysanoptera members at the mitochondrial level. The complete mitochondrial genome of *F. serrata* is 16,596 bp in length and includes 13 protein-coding genes (PCGs), 22 transfer RNA (tRNA) genes, 2 ribosomal RNA (rRNA) genes, and 1 noncoding control region. A+T accounted for 75% of the total bases in the mitochondrial genome of *F. serrata*, revealing an obvious AT bias. Among the 13 PCGs, except for *nad5*, which had a start codon of TTG, the remaining genes had ATNs typical of insects (ATA, ATT, ATC, and ATG); *nad1*, *nad2*, *nad3*, and *atp8* had incomplete termination codons of TA or T. The remaining nine PCGs were complete with the termination codon TAA. Of the 22 tRNA secondary structures, all were typical cloverleaf secondary structures except for *trnS1*, which was missing the DHU arm. Compared with the hypothetical ancestral gene arrangement of arthropods, *F. serrata* presented extensive gene rearrangement, with 23 translocated genes, 8 inverted genes, and 5 shuffled genes. Both maximum likelihood (ML) and Bayesian inference (BI) phylogenetic trees resulted in similar topologies: ((Thripidae + (Stenurothripidae + Aeolothripidae)) + Phlaeothripidae), with Thripidae, Aeolothripidae and Phlaeothripidae being monophyletic groups, whereas *F. serrata* is closely related to *Thrips palmi*, and the two are sister groups.

## 1. Introduction

Mitochondria are semiautonomous organelles that are ubiquitously expressed in insect cells and play important roles in the regulation of cellular metabolism, senescence, apoptosis, and disease [1]. The insect mitochondrial genome is a double-stranded, closed-loop molecule that is typically 14–21 kb in size, and it contains 37 genes (22 tRNAs, 2 rRNAs, and 13 PCGs) as well as one noncoding region (AT-rich region) associated with transcription and replication initiation [1,2]. The mitochondrial genome is characterized by its small size and high copy number, and exhibits direct gene homology, infrequent recombination, maternal inheritance, and a rapid evolutionary rate [1]. At present, mitochondrial genomes have been widely used in molecular evolution and phylogenetic analysis, rapid identification, population genetics, and other studies of insects [3,4,5,6].

The order Thysanoptera (commonly known as thrips) contains two suborders: Terebrantia and Tubulifera. There are 6415 species of 787 genera in 9 families worldwide [7], and 699 species and 169 genera are known in China [8]. Thrips have a wide variety of species and complex feeding habits, including phytophagous, mycophagous, and predatory habits. Types of phytophagous thrips include noxious thrips, which are harmful to crops, and beneficial thrips, which are weed-control agents; some predatory thrips are important natural enemies of some small insects, and mycophagous thrips, as important decomposers in the ecosystem, play an important role in the energy cycle of the biosphere [9]. Therefore, accurate identification of thrips species and understanding the phylogenetic relationships of these insects are important references for thrips control and utilization of economic germplasm resources. However, the sequencing of the mitochondrial genome of Thysanoptera insects has only recently been completed, and studies on the mitochondrial genome of thrips have been limited because the mitochondrial structure of thrips is drastically rearranged, with an obvious tendency toward accelerated evolution and having multiple control regions and cleavage phenomena, which makes it difficult to amplify the mitochondrial genome using universal primers [10]. The phylogenetic position of the order Thysanoptera is mainly based on morphological characters or a few gene constructs, and its classification within the order needs further study.

*F. serrata*, also known as cane ventral tooth thrips or cane brown thrips, belongs to the order Thysanoptera, suborder Terebrantia, family Thripidae, and genus *Fulmekiola*. It is the only species in the genus *Fulmekiola*, and this thrips is a common leaf pest that infests sugarcane [11,12]. *F. serrata* originated in Asia and was first observed on sugarcane in Java. *F. serrata* have also been documented in China, Japan, Vietnam, Pakistan, Bangladesh, India, the Philippines, Malaysia, and Indonesia, and they have now spread to Mauritius, Réunion, Madagascar, Trinidad and Barbados, Venezuela, and South Africa [13,14]. In 2017, United States (U.S.) Pest Alerts reported the discovery of *F. serrata* in southern Florida and noted its rapid expansion and spread [15]. *F. serrata* also occurs in Yunnan, Guangxi, Guangdong, Hainan, Fujian, Taiwan, Guizhou, Sichuan, Hunan, Jiangsu, Zhejiang, and other provinces and regions in China [16].

*F. serrata* mainly infests the new leaves and the tails of sugarcane leaves, and the nymphs and adults gather in the unfolded new leaves to suck the sap. The infested leaves are slightly water-logged with yellow spots when they are not unfolded, and after the leaves are unfolded, they have yellow or light-yellow patches. When the damage is serious, the leaves will be curled up and shriveled, twisted and knotted, and even dried up and dead, affecting the photosynthesis of the leaves, hindering the growth of sugarcane, and causing a reduction in yield [17]. According to the survey, the damage by *F. serrata* could cause a reduction of 18.00% to 26.80% in sugarcane and 16.20% to 24.00% in sucrose in South Africa [18]. In Yunnan, the yield loss of more resistant varieties ranged from 13.04% to 21.06% and the loss of sugar from 8.33% to 8.40%, while yield loss of less resistant varieties was as high as 40.58% and the loss of sugar was 8.44% [19].

Current research on *F. serrata* has focused on several aspects of biology, ecology, and control methods [12,15,20,21,22,23,24], but there have not been any in-depth studies on the basic characteristics and phylogeny of the mitochondrial genome of *F. serrata.* In this study, the whole mitochondrial genome sequence of *F. serrata* was determined, and the mitochondrial genome sequences of 17 species of Thysanopteran insects included in GenBank were used in comparative analysis to explore the phylogenetic relationships among families, genera, and species of Thysanoptera. The goal of the work was to lay a foundation for the study of relevant population genetics, the molecular ecology of *F. serrata*, and the molecular phylogeny of Thripidae.

## 2. Results

### 2.1. Mitochondrial Genome Composition

**Figure 1:**
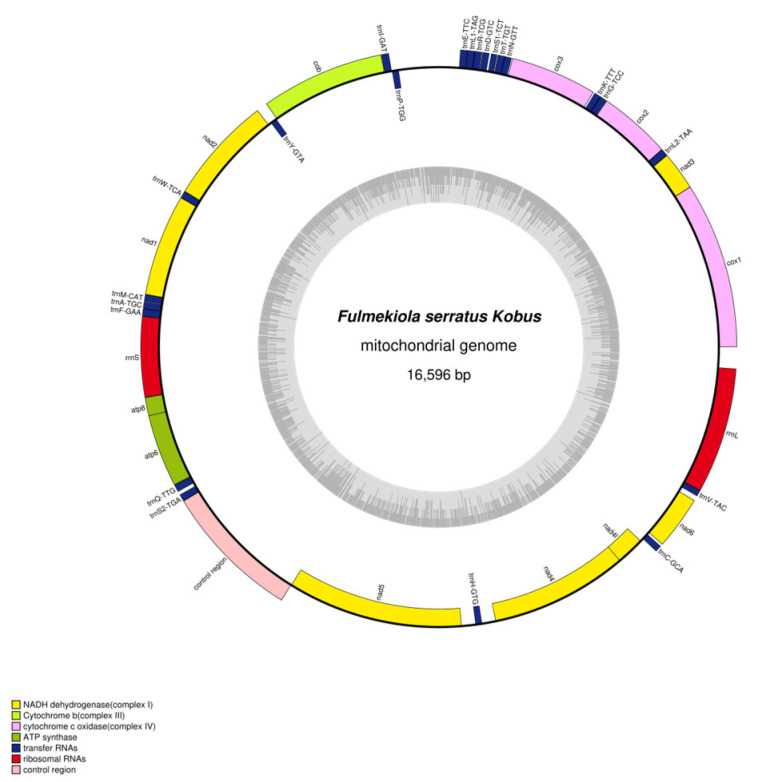
Mitochondrial genome structure of *F. serrata*.

The mitochondrial genome of *F. serrata* is 16,596 bp in length, the GenBank accession number is PP861183, the A+T content is 75%, and the G+C content is 25%. It contains 13 PCGs, 22 tRNA genes, 2 rRNA genes (*rrnL* and *rrnS*), and 1 noncoding control region (Figure 1). The J-strand (major strand) encodes 31 genes, including 10 PCGs, 19 tRNA genes, and 2 rRNA genes; the N-strand (minor strand) encodes 6 genes, including 3 PGCs and 3 tRNA genes. The noncoding control region is located between *trnS2* and *nad5*; it has a length of 1250 bp, A+T content of 73.52%, and G+C content of 26.48% (Table 1).

Among the 37 genes in the coding region of the mitochondrial genome of *F. serrata*, there are areas of overlapping genes or gene spacers between neighboring genes. There are 6 overlapping regions, ranging from 1–7 bp, with the longest overlap being 7 bp between *atp6* and *trnQ* and between *nad4l* and *trnC.* There are 19 gene spacers, ranging from 1–570 bp, with the longest spacer being 570 bp between *trnP* and *trnI*. There are 12 regions with neither overlap nor spacer (Table 1).

### 2.2. Analysis of Protein-Coding Genes

The length of the 13 PCGs of the mitochondrial genome of *F. serrata* is 10,895 bp, of which the longest gene was *nad5*, with a length of 1,683 bp, and the shortest gene was *atp8*, with a length of 169 bp; they are located in the N and J strands, respectively. The A+T content of the 13 PCGs is 74.22%, with an AT skew of −0.144 and a GC skew of −0.081 (Table 2). There are 12 genes with ATN as the start codon, among which *nad3*, *cox2*, *cox3*, *cob*, *nad2*, *nad1*, and *nad6* have ATA as the start codon; *atp8*, *atp6*, and *nad4* have ATT as the start codon; *cox1* and *nad4l* have ATC and ATG as the start codon; and *nad5* has TTG as the start codon. The genes *nad3*, *nad2*, and *atp8* have an incomplete termination codon of a single T, *nad1* has an incomplete termination codon of TA, and the remaining nine PCGs have a termination codon of TAA (Table 1).

**Figure 2:**
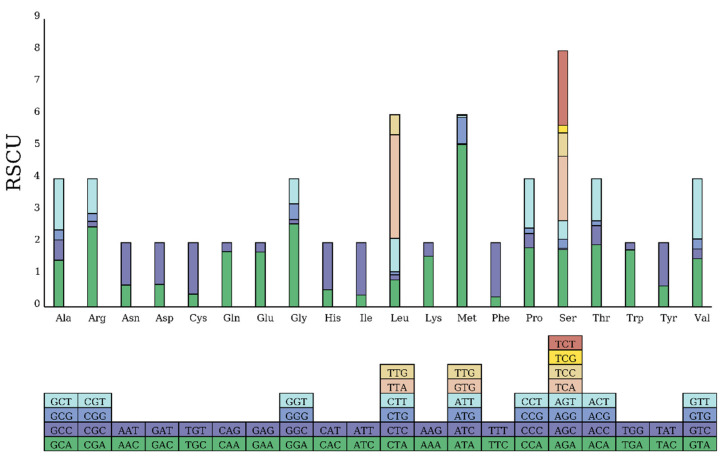
Relative synonymous codon usage (RSCU) in the mitochondrial genome of *F. serrata.* The box below the bar chart represents all codons encoding each amino acid, and the height of the column above represents the sum of all RSCU values.

The 13 PCGs of the *F. serrata* mitochondrial genome encode a total of 3634 codons (including the stop codon), and the three most frequent codons are UUU (phenylalanine, Phe), AUU (isoleucine, Ile), and UUA (leucine, Leu), which are found with total frequencies of 398, 306, and 270, respectively. UAG and GUG were not observed (Figure 2). The most frequent amino acids in protein-coding genes are Leu (13.76%), Phe (12.93%), and Ser (10.95%). Relative synonymous codon usage (RSCU) showed a large difference in RSCU values among codons encoding the same amino acid, indicating an obvious bias in codon usage frequency in the mitochondrial genome of *F. serrata*.

### 2.3. tRNA Gene and rRNA Gene

The mitochondrial genome of *F. serrata* contains 22 tRNA genes with a length of 1399 bp. These tRNA genes account for 8.43% of the mitochondrial genome of *F. serrata*, and the length of individual tRNA genes ranges from 56–68 bp. Nineteen tRNA genes were located in the J chain and three tRNA genes were located in the N chain. The tRNA genes contain an A+T content of 79.41%, an AT skew of 0.073, and a GC skew of 0.035. The J chain of the *F. serrata* mitochondrial genome contains 2 rRNA genes, *rrnS* and *rrnL*, with lengths of 722 and 1136 bp, respectively, which are located in *trnF* and *atp8* (*rrnS*) and *trnV* and *cox1* (*rrnL*). The length of rRNA genes in the mitochondrial genome of *F. serrata* is 1858 bp, with an A+T content of 78.53%, an AT skew of 0.19, and a GC skew of 0.033 (Table 2).

**Figure 3:**
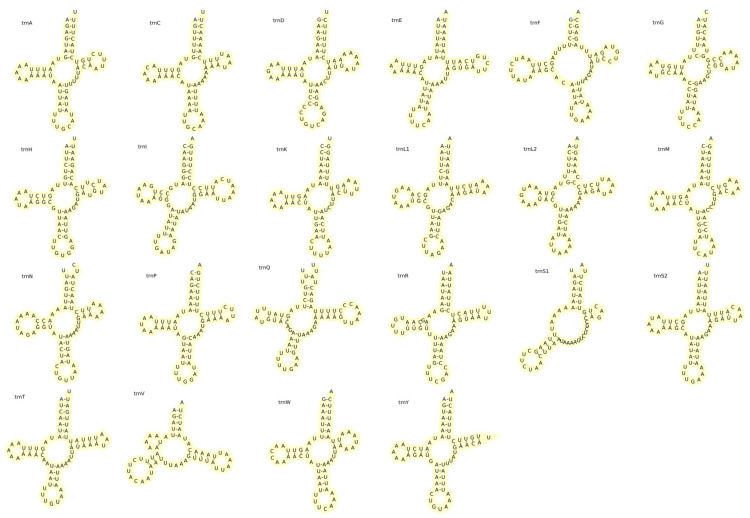
Secondary structure of 22 tRNA genes in the mitochondrial of *F. serrata*.

Among the 22 tRNA genes in the mitochondrial genome of *F. serrata*, all 21 tRNAs present a typical cloverleaf structure, except for *trnS1*, which lacks the DHU arm. Eight pairs of mismatched bases appear in the secondary structure of the twenty-two tRNA genes of the *F. serrata* mitochondrial genome, and these bases are found in the amino acid acceptor arm and antisense codon arm of *trnA*, the variable loops of *trnD* and *trnL1*, the DHU arm of *trnG*, the amino acid acceptor arm of *trnI*, and the antisense codon arm and the DHU arm of *trnQ*. With the exception of the mismatched bases on the variable loop of *trnD*, which is an A-C pair, all mismatched bases are G-U (Figure 3).

### 2.4. Gene Arrangement

The mitochondrial gene arrangement is relatively conserved in insects, and the mitochondrial gene arrangement of *Drosophila yakuba* is considered the ancestor of arthropods [2,25]. Based on the positional changes of genes following gene rearrangements, mitochondrial gene rearrangements can be categorized into three types: translocation, inversion, and shuffling [26]. Compared with the hypothetical arthropod ancestor gene arrangement, here, we found that 23 genes are shifted, 8 genes are inverted, and 5 genes are shuffled in the mitochondrial genome of *F. serrata* (Figure 1). These rearrangements include 11 PCGs, 18 tRNA genes, 2 rRNA genes, and 1 noncoding control region; both *rrnS* and *rrnL* code in the reverse order of the ancestral sequence, 5′ to 3′ compilation and far apart, while the control region moved into an adjacent position. In addition, the gene clusters *trnL2*-*cox2*, *nad2*-*trnW*, *atp8*-*atp6*, *nad5*-*trnH*-*nad4*-*nad4l*, and *trnV*-*rrnL* were conserved in the mitochondrial genome of *F. serrata*. With the exception of the gene cluster *trnV*-*rrnL*, which is in the opposite order of the ancestral gene arrangement of the hypothetical arthropods, the gene clusters were in the same order as the pseudo-arthropod ancestral gene arrangement.

### 2.5. Analysis of Phylogenetic

**Figure 4:**
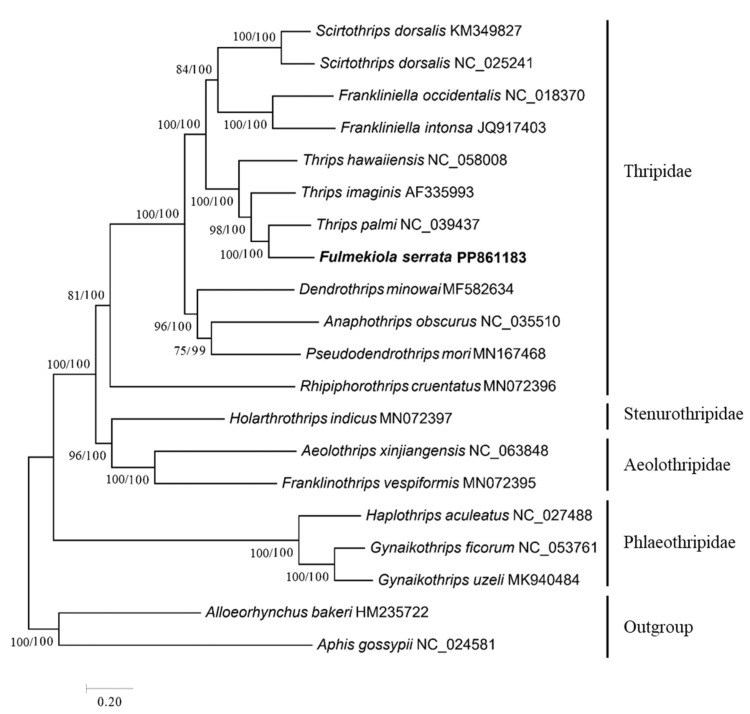
Phylogenetic tree from the nucleotide sequences of 13 protein-coding genes constructed based on the maximum likelihood method and the Bayesian method.

The phylogenetic trees of 17 species of thrips from 4 families of 2 suborders of Thysanoptera were constructed using ML and BI (Figure 4). The topologies of the phylogenetic trees constructed by the two methods were identical, and the phylogenetic relationship is as follows: ((Thripidae + (Stenurothripidae + Aeolothripidae)) + Phlaeothripidae), which supported the notion that Thripidae, Aeolothripidae, and Phlaeothripidae were monophyletic groups. Thripidae, Stenurothripidae, and Aeolothripidae were clustered into a single unit, which formed a sister group with Phlaeothripidae. Stenurothripidae and Aeolothripidae were sister groups and formed a branch with Thripidae. Among the Thripidae, *F. serrata* and *T. palmi* were more closely related, and they were sister groups. The node support values of the ML phylogenetic tree and BI phylogenetic tree on this branch were 100 and 100, respectively.

## 3. Discussion

In this study, we obtained the whole mitochondrial genome sequence of *F. serrata* using high-throughput sequencing technology; it has a total length of 16,596 bp, consisting of 37 genes and a noncoding control region, which was similar to the reported mitochondrial genome of Thysanopteran insects in terms of features such as gene structure, codon use of PCGs, and the secondary structure of tRNAs [10]. The mitochondrial genome sequence of *F. serrata* has an AT content of 75% and a GC content of 25%, and this significant difference reveals that *F. serrata* has a high AT bias, which is similar to that of other Thysanopteran insects [10]. The AT skew is 0.128 and the GC skew is −0.12, which is similar to that of the metazoan mitochondrial genome, which has positive AT and negative GC skewness [27].

Among the PCGs, all 12 start codons of the *F. serrata* mitochondrial genome are ATN, which is typical for insects; only the start codon of *nad5* is TTG. The termination codon of most of the PCGs is the typical TAA, and only *nad1*, *nad2*, *nad3*, and *atp8* have incomplete TA or T as the termination codon. This phenomenon is also present in other thrips mitochondrial PCGs, such as the *Megalurothrips usitatus* mitochondrial genome, where most of the PCG start codons are ATNs; only *ND4* has a TTG start codon, and *nd1*, *nd2*, *nd4*, and *atp8* end with an incomplete termination codon T [28]. Incomplete stop codons are also common in other insect genomes, and they are thought to be recovered by post-transcriptional polyadenylation [10,25].

In terms of tRNA structure, the 21 tRNA secondary structures of the mitochondrial genome of *F. serrata* have a typical cloverleaf structure, with only trnS1 missing the DHU arm. This phenomenon is prevalent in the mitochondrial genomes of Thysanopteran insects, where *trnS1* lacks the DHU arm [6]. In addition, *trnA* and *trnV* of *Anaphothrips obscurus* lack the complete DHU arm, and *trnV* of *Thrips imagines*, *Frankliniella occidentalis*, and *Frankliniella intonsta* all lack the complete DHU arm [10].

The mitochondrial genome of *F. serrata* has only one noncoding control region, unlike other thrips that have two or three control regions. For example, *Thrips imaginis*, *Scirtothrips dorsalis EA*, *T. palmi*, *Neohydatothrips samayunkur*, *Franklinothrips vespiformis*, *Thrips hawaiiensis*, *Taeniothrips tigris*, *Aeolothrips xinjiangensis*, *Aeolothrips indicus*, *Stenchaetothrips biformis*, *Thrips parvispinus*, and *Psephenothrips eriobotryae* have two control regions [6,29,30,31,32,33,34], and very few thrips, such as *F. occidentalis*, *Frankliniella intonsa*, *S. dorsalis SA*, and *Aptinothrips stylifer* have three control regions in the mitochondrial genome [6,35]. The location and length of the individual control regions varied, which is probably due to duplications and gene rearrangements, and the control regions of the mitochondrial genome of thrips vary in number, size, and genomic location [6].

The thrips mitochondrial genome is characterized by a high rate of gene rearrangements, control region duplications, and tRNA mutations [36]. However, PCGs and rRNA genes in the mitochondrial genome of *F. serrata* are arranged in a more conservative order, similar to those of *F. intonsa*, *F. occidentalis*, *T. imagines*, *S. dorsalis EA1*, *T. palmi*, and *N. samayunkur* [10]; however, the order of the tRNA genes is less predictable, suggesting that insect mitochondrial tRNA genes have a greater degree of mobility than PCGs and rRNA genes [37]. The mitochondrial genome of *F. serrata* is highly rearranged, with a total of 36 genes shifted, inverted, or shuffled, which is most plausibly explained by recombination within the mitochondrial genome [38].

In this study, we constructed phylogenetic trees of the representative species of Thysanoptera using ML and BI, and the results of the two phylogenetic tree analysis methods support the idea that Thripidae is a monophyletic group, which is consistent with the results from a previous report [39], which was phylogenetic study of 70 genera of Thripidae based on 5 gene fragments. In Thripidae, *F. serrata* and *T. palmi* are more closely related and are sister groups, but *F. serrata* is in the genus *Fulmekiola* and *T. palmi* is in the genus *Thrips*. This phenomenon of sister groups of thrips in different genera of the same family indicates that they share a recent common ancestor and maintain relatively independent development paths during evolution. Although they belong to different genera, they are very closely related and together form part of the thrips family [40]. This also suggests that the evolutionary relationships between different genera within the family Thripidae are complex, and further research is needed to clarify them.

## 4. Materials and Methods

### 4.1. Insect Sample Collection and DNA Extraction

Adult *F. serrata* species were collected from the First Research Experimental Base of Sugarcane Research Institute, Yunnan Academy of Agricultural Sciences, in May 2023. The collected samples were stored in 100% ethanol and preserved in an ultralow-temperature refrigerator of −80 °C at the Entomology Laboratory of Sugarcane Research Institute, Yunnan Academy of Agricultural Sciences. The 50 *F. serrata* DNA samples were extracted according to the instruction manual for the animal genomic DNA Extraction Kit (Sangon Biotech (Shanghai) Co., Ltd., Shanghai, China). The quality and concentration of the DNA samples was detected by a NanoDrop 2000 spectrophotometer and 1% agarose gel electrophoresis, respectively.

### 4.2. High-Throughput Sequencing and Mitochondrial Genome Assembly

The qualified DNA samples were sent to Genepioneer Biotechnologies Co., Ltd. (Nanjing, China) for the construction of small fragment libraries, and the constructed small fragment libraries were subjected to paired-end high-throughput sequencing using an Illumina NovaSeq 6000 sequencing platform with a sequencing length of 150 bp. Fastp v0.23.4 software was used to cut and filter the quality of the raw data obtained from sequencing, truncate the sequencing junctions and primer sequences in the reads, and filter out the reads with average quality and average quality values less than Q5 and reads with N content greater than 5 to obtain the clean data dataset [41,42]. Mitochondrial genome assembly and splicing of the clean dataset were performed using SPAdes v3.10.1 software [43], resulting in high-quality mitochondrial genome sequences.

### 4.3. Mitochondrial Genome Annotation and Analysis

Mitos2 (http://mitos2.bioinf.uni-leipzig.de/ (accessed on 22 September 2024)) was used to annotate the mitochondrial genome of *F. serrata* and predict the secondary structure of tRNA [44]. The annotation results were confirmed by comparison with homologous sequences in the NCBI database, and the results were subsequently submitted to the NCBI. The mitochondrial genome of *F. serrata* was mapped via OGDRAW v1.3.1 (https://chlorobox.mpimp-golm.mpg.de/OGDraw.html) [45]. MEGA-X software (accessed on 22 September 2024) was used to analyze and calculate the base composition and codon usage frequency of all protein-coding genes in the mitochondrial genome of *F. serrata* [46]. The formulas AT skew = (A − T))/(A + T) and GC skew = (G − C)/(G + C) were used to calculate AT skew and GC skew, respectively.

### 4.4. Phylogenetic Analysis

Phylogenetic trees were constructed via ML and BI methods based on the newly sequenced mitochondrial genome of *F. serrata* and the nucleotide sequences of 13 protein-coding genes in the mitochondrial genomes of 17 species of Thysanopteran insects reported in GenBank; *Alloeorhynchus bakeri* (Hemiptera: Nabidae) and *Aphis gossypii* (Hemiptera: Aphididae) were used as outgroups. ML analysis was performed via RAxML v8.2.10 (https://cme.h-its.org/exelixis/software.html (accessed on 22 September 2024)) software, and the GTRGAMMA model was chosen to construct the phylogenetic tree via the self-expansion method (bootstrap) (1000 repetitions) to test the confidence of the branching nodes. MrBayes v3.2.7a (http://nbisweden.github.io/MrBayes/ (accessed on 22 September 2024)) software was utilized for BI analysis, and the GTR+I+G model was selected for the work via four Markov chain Monte Carlo (MCMC) methods running simultaneously for 2,000,000 generations. Sampling took place every 100 generations, and 25% of the aged samples were discarded to construct the BI phylogenetic tree.

## 5. Conclusions

*F. serrata* is the only species in the genus *Fulmekiola*, and its complete mitochondrial genome sequence is the first to be sequenced in that genus. We sequenced the complete mitochondrial genomic sequences of *F. serrata* for the first time in this study. This study will make a significant contribution to our understanding of the phylogenetic relationships of the higher-order elements of Thysanoptera members at the mitochondrial level. The complete mitochondrial genome of *F. serrata* is 16,596 bp, and its mitogenome contains 13 PCGs, 22 tRNAs, 2 rRNAs, and 1 non-coding region. Phylogenetic relationship analysis showed that Thripidae, Aeolothripidae, and Phlaeothripidae are monophyletic groups, and *F. serrata* and *T. palmi* are sister groups. These findings provide critical insights into the genetic makeup and evolutionary history of *F. serrata* and suggest useful molecular information on the phylogenetic and evolutionary relationships the phylogenetic relationships among families, genera, and species of Thysanoptera.

## Figures and Tables

**Table 1:** Annotation of the mitochondrial composition of *F. serrata*.

Gene	Strand	Location	Size (bp)	Anticodon	Start Codon	Stop Codon	Intergenic Nucleotides
*cox1*	J	1–1500	1500	-	ATC	TAA	192
*nad3*	J	1497–1848	352	-	ATA	T--	−4
*trnL2*	J	1849–1913	65	TAA			0
*cox2*	J	1914–2570	657	-	ATA	TAA	0
*trnG*	J	2572–2634	63	TCC			1
*trnK*	J	2634–2695	62	TTT			−1
*cox3*	J	2708–3490	783	-	ATA	TAA	12
*trnN*	J	3497–3560	64	GTT			6
*trnT*	J	3558–3622	65	TGT			−3
*trnS1*	J	3627–3682	56	TCT			4
*trnD*	J	3698–3761	64	GTC			15
*trnR*	J	3765–3830	66	TCG			3
*trnL1*	J	3829–3893	65	TAG			−2
*trnE*	J	3894–3958	65	TTC			0
*trnP*	N	4529–4592	64	TGG			570
*trnI*	J	4595–4662	68	GAT			2
*cob*	J	4664–5779	1116	-	ATA	TAA	1
*trnY*	N	5784–5846	63	GTA			4
*nad2*	J	5874–6846	973	-	ATA	T--	27
*trnW*	J	6847–6909	63	TCA			0
*nad1*	J	6910–7829	920	-	ATA	TA-	0
*trnM*	J	7830–7892	63	CAT			0
*trnA*	J	7895–7957	63	TGC			2
*trnF*	J	7958–8024	67	GAA			0
*rrnS*	J	8025–8746	722	-			0
*atp8*	J	8747–8915	169	-	ATT	T--	0
*atp6*	J	8909–9568	660	-	ATT	TAA	−7
*trnQ*	J	9569–9636	68	TTG			0
*trnS2*	J	9667–9730	64	TGA			30
D-loop	J	9731–10,980	1250	-			0
*nad5*	N	10,981–12,663	1683	-	TTG	TAA	0
*trnH*	N	12,795–12,857	63	GTG			131
*nad4*	N	12,987–14,306	1320	-	ATT	TAA	129
*nad4l*	N	14,300–14,575	276	-	ATG	TAA	−7
*trnC*	J	14,603–14,664	62	GCA			27
*nad6*	J	14,684–15,169	486	-	ATA	TAA	19
*trnV*	J	15,213–15,268	56	TAC			43
*rrnL*	J	15,269–16,404	1136	-			0

Note: J refers to major strand, N refers to minor strand. Gene overlap is represented by a negative number, gene interval is represented by a positive number, and 0 means no gene interval and overlap.

**Table 2:** Nucleotide composition and skewness of the mitochondrial genome of *F. serrata*.

*Fulmekiola serrata*	Size (bp)	A%	T%	G%	C%	A+T%	G+C%	AT-Skew	GC-Skew
Whole mitogenome	16,596	42.28	32.72	11	14	75	25	0.128	−0.12
PCGs	10,895	31.75	42.47	11.85	13.93	74.22	25.78	−0.144	−0.081
tRNAs	1399	42.6	36.81	10.65	9.94	79.41	20.59	0.073	0.035
rRNAs	1858	46.72	31.81	11.09	10.39	78.53	21.47	0.19	0.033
Control region	1250	41.68	31.84	10.96	15.52	73.52	26.48	0.134	−0.172

## Data Availability

All of the data that support the findings of this study are available in the main text. The genome sequence data are openly available in GenBank of NCBI at https://www.ncbi.nlm.nih.gov/, accessed on 22 September 2024 under accession no. PP861183.
